# Trends in Opioid Use in a Cohort of Patients with Rheumatoid Arthritis

**DOI:** 10.1155/2020/3891436

**Published:** 2020-07-16

**Authors:** Manuel E. Machado-Duque, Diana Marcela Ramírez-Valencia, María Mónica Murillo-Muñoz, Jorge E. Machado-Alba

**Affiliations:** ^1^Grupo de Investigación en Farmacoepidemiología y Farmacovigilancia, Universidad Tecnológica de Pereira-Audifarma S.A, Pereira, Colombia; ^2^Grupo Biomedicina, Fundación Universitaria Autónoma de Las Américas, Pereira, Colombia

## Abstract

The objective was to determine the trend in the use of opioid analgesics in a cohort of patients diagnosed with and treated for rheumatoid arthritis (RA) in 24 cities in Colombia. This retrospective cohort study included adult patients diagnosed with RA, which was managed in a specialized institution in Colombia between January 2011 and December 2012. The first rheumatology visit was recorded as an index date, and monthly monitoring of the analgesic medication received was performed until December 2017. Sociodemographic variables, the use of opioids, and concomitant prescriptions were evaluated. A total of 1,329 patients diagnosed with and treated for RA were included; they had a mean age of 61.2 ± 11.8 years and were predominantly females (*n* = 936; 82.9%). A total of 1,129 (84.9%) subjects used opioids for at least one month, and a growing trend, from 13.5% to 21.4%, was observed in patients who received opioids every month throughout a 7-year follow-up of the cohort. In total, 46.7% of the cases used opioids for more than 12 months. The most commonly used opioids were codeine (76.3%) and tramadol (71.1%). All patients received conventional disease-modifying antirheumatic drugs (DMARDs), 85.6% received systemic corticosteroids, 73.9% received nonsteroidal anti-inflammatory drugs, and 15.9% received biological DMARDs. A high proportion of opioid use was shown for pain management in patients with RA, in many cases for more than 12 months, in whom the efficacy and especially safety, related to the risk of dependence, should be monitored.

## 1. Introduction

Currently, rheumatoid arthritis (RA) affects the adult population, with an estimated prevalence between 0.5% and 1% and an incidence of 5–50 per 100,000 population, mainly affecting women over 65 years. RA is a chronic, progressive autoimmune disease characterized by persistent synovial and systemic inflammation associated with joint destruction, whose main symptom is chronic joint pain, which is the most common reason for consultation [1, 2].

For the treatment of pain caused by the RA activity, there are multiple classes of drugs, such as opioids. Although the efficacy and relative safety of these drugs have been previously established for short-term use, there is limited evidence regarding their safety upon chronic use [3, 4]. In recent decades, a significant increase in opioid use has become a concern in Europe, Canada, and the United States [5, 6], in particular for their narrow therapeutic margin, tolerance, dependence, and adverse events such as delirium, somnolence, and falls [7–9].

People affected by RA pain, along with patients with neoplasm-related pain, have traditionally been treated with opioids. However, recent studies have shown an increase in opioid use, mainly in the first group [10, 11]. In addition, the latter group presents an increased risk of long-term use, situation that can lead to related problems such as dependency, addiction, and overdose, as well as an increased risk of death in this population; therefore, the need to rationalize the use for short periods and in patients with indications where the benefit outweighs the risk associated with opioids has been identified [10].

The Colombian Health System offers universal coverage through two enrollment regimes, one paid for the employer and employee and another subsidized by the state, which has a list of drugs included in a benefit plan for all patients and containing different analgesics, antirheumatics, and corticosteroids. Given the risks and adverse events that may arise from their chronic use, it was proposed to determine the trend in the use of opioid analgesics in a cohort of patients diagnosed with and treated for RA in 24 cities in Colombia.

## 2. Materials and Methods

An observational retrospective cohort study was conducted, which included all patients with diagnosis of RA, according to the codes of the International Classification of Diseases version 10.0 (ICD-10), and older than 18 years treated with conventional or biological DMARDs (access to DMARDs in Colombia is fully covered by the Health System) at the Specialized Healthcare Provider Institution (Institución Prestadora de Servicios Especializada, IPS-E) in 24 cities in Colombia (Ithe rheumatologists of IPS Especializada, use EULAR criteria for the diagnosis of RA), who were affiliated with seven different insurers or EPS (Entidades Promotoras de Salud). Patients who received RA care between January 2011 and December 2012 were included, and the index date for the start of follow-up was defined as the date of the first specialized consultation within the observation period (the use of medications of interest was the follow-up month by month). Medications dispensed until December 2017 were identified, and patients with a follow-up of less than 6 months and those who were prescribed opioids in the month prior to the index date were excluded.

For the included cases, the medications dispensed by Audifarma, which is the logistics operator in charge of medication delivery to these patients, were determined from its database of drug use by more than 6.5 million people, a database that is widely used in pharmacoepidemiological studies.

A database with the information on each subject was created, which was reviewed by two of the authors and validated by another one and included the following variables:

1. Sociodemographic and clinical: age, sex, city of residence, EPS insurer, and diagnosis according to ICD-10.
2. Use of opioids: prescribed drugs: opioid medication classified by international name and partial or total agonist capacity (weak opioids: codeine, dihydrocodeine, hydrocodone, tramadol, and tapentadol; strong opioids: fentanyl, morphine, oxycodone, hydromorphone, and methadone), date of prescription, dose, duration of use of the drug (in months), change in therapy, increased dose, and use of combined opioids. The use of opioids was defined as a prescription of any opioid during the follow-up period. Chronic use was defined as a prescription for 60 or more days at usual doses, and prolonged use was categorized as a continuous prescription for more than 1 year.
3. Coprescriptions: combined prescriptions drugs of the following groups of medications were identified as follows (on the index date): (a) conventional disease-modifying antirheumatic drugs (DMARDs), (b) biological DMARDs, (c) nonsteroidal anti-inflammatory drugs (NSAIDs), (d) acetaminophen, (e) dipyrone, (f) systemic corticosteroids, (g) antidepressants (tricyclic, selective serotonin reuptake inhibitors (SSRIs), dual-action, and atypical), (h) antiepileptic drugs, and (i) antipsychotics.

The protocol was approved by the Bioethics Committee of the Universidad Tecnológica de Pereira in the category of “research without risk” and waived the authors from obtaining informed consent; the principles of confidentiality established by the Declaration of Helsinki were respected, and in no case was the personal data of the patients considered. This is not an experimental study, and there was no intervention on patients; so, it was not necessary to obtain informed consent according to Resolution ^#^8430 of 1993 of the Ministry of Health of Colombia.

### 2.1. Analysis Plan

The database was created in Microsoft Excel 2016 for Windows. Analyses were performed using the statistical package SPSS 24.0 (IBM, New York, NY, USA). The frequencies and proportions of categorical variables and measures of central tendency and dispersion of quantitative variables were determined by univariate analysis according to their normality (Kolmogorov–Smirnov test). Bivariate analyses were performed using the *χ*^2^. A model to adjust was used with binary logistic regression to identify variables that were associated with the prescription of opioids, including those variables with plausibility to explain the outcome and those with statistically significant differences (*p* < 0.05) in the bivariate analysis in the dataset with the use of opioids for more than 12 months. A *p* value <0.05 was established as statistically significant.

## 3. Results

From an original cohort of 1,911 subjects with RA who were followed, 1,329 patients who were diagnosed with RA and treated with conventional or biological DMARDs were identified and followed-up at IPS-E during the period of initial observation. The rest were excluded because their follow-up started after the observation period or was less than 6 months. A total of 1,129 (84.9%) patients used opioids at least once since prescribed as a therapy at any time during the follow-up (mean: 85.7 months; range: 66–90 months); these patients had a mean age of 61.2 ± 11.8 years and were predominantly women (*n* = 936; 82.9%) (Table 1). The patients were from 24 different cities, most frequently from Bogotá (*n* = 773; 68.5%), Pereira (*n* = 95; 8.4%), Manizales (*n* = 84; 7.4%), Medellin (*n* = 58; 5.1%), and Cali (*n* = 30; 2.7%).

**Figure 1:**
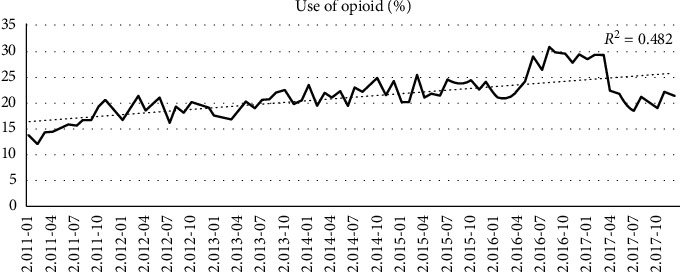
Trend in opioid use in a cohort of 1,329 patients with rheumatoid arthritis in Colombia.

On an average in the follow-up, 21.2% of all patients had a prescription of opioid analgesics every month, with the proportion ranging from 13.5% (*n* = 179) in January 2011 to 21.4% (*n* = 282) at the end of December 2017.  Figure 1 shows the trend in the use of opioids. The mean duration of the first opioid prescription was 38.1 ± 21.5 days (range: 21–327). In total, 46.7% (*n* = 528) of the patients used opioids for more than 12 months during the observation period (opioid use did not have to be continuous during follow-up).

We identified 416 patients (36.8%) who used a single opioid analgesic during the observation period, 434 (38.4%) who used two different opioids, 229 (20.3%) with three opioids, and 43 (3.8%) who received up to four different opioids. The most used opioids were codeine and tramadol; Table 2 shows the prescription patterns for the opioid analgesics used.

Regarding the management of RA, it was found that all patients were managed with conventional DMARDs and 179 (15.9%) with biological DMARDs. In addition, 966 patients (85.6%) used systemic corticosteroids, and 834 (73.9%) received nonsteroidal anti-inflammatory drugs (NSAIDs). Furthermore, the use of other concomitant medications, such as acetaminophen (*n* = 882; 78.1%), antidepressants (*n* = 412; 36.5%), anticonvulsants (*n* = 126; 11.2%), benzodiazepines (*n* = 42; 3.7%), and antipsychotics (*n* = 12; 1.1%), was observed.

### 3.1. Multivariate Analysis

The multivariate analysis indicated that patients older than 45 years and those who received antidepressants, antiepileptic drugs, benzodiazepines, biological DMARDs, and systemic corticosteroids were more likely to receive opioid analgesics for a period longer than 12 months, while no variable was found that would reduce this probability (see Table 3).

## 4. Discussion

Opioids have been studied in the coadjuvant management of pain in multiple pathologies, including RA. However, their benefits are unknown upon chronic use because of limited evidence regarding the opioid safety and efficacy in these cases [4, 12]. In this study, a growing trend could be observed in the use of opioids by patients with RA in Colombia. In addition, almost half of the patients received opioids for more than a year, a situation that highlights the risks associated with this therapy, especially in the long-term, and is considered epidemic even in developed countries [13–15].

A study by Lee et al. has shown that the use of opioid analgesics by RA patients doubled in the Consortium of Rheumatology Researchers of North America (CORRONA) cohort in the United States, having increased from 7.4% in 2002 to 16.9% in 2015 [16]. A study by Zamora-Legoff et al., also in the United States, which reviewed the chronic use of this group of drugs in patients with RA, found after 10 years of follow-up that 40% of patients was prescribed with an opioid at least once [10]. Meanwhile, it was found in our study, with an average follow-up of 7 years, that 84% of the subjects received opioids at least once, probably because of less restriction in their use in this cohort.

The same study by Zamora-Legoff et al. found that 12.0% of patients with RA received opioid analgesics for more than 60 days, which was considered a chronic use, while in this study, 87.6% of the subjects received opioids for the same period of time, and 46.7% used the drugs for more than a year [10], a worrisome situation considering the lack of evidence on opioid efficacy and long-term safety. In fact, there is no published controlled clinical trial that has studied the use of opioids in patients with RA for more than 8 weeks. Although the results of these short-term studies have shown better outcomes than those observed with placebo in terms of the analgesic efficacy, a higher incidence of adverse events has been reported [4, 17]. For this reason, most clinical practice guidelines recognize the problem of the long-term use of opioids, as well as the lack of clinical evidence and the need to limit the use and potential risks of opioids, posing limitations on the doses used, advising on dose titration precautions, and promoting awareness on the dangers associated with the use of fentanyl patches and methadone [18].

There is a lack of published information about chronic noncancer pain in Colombia. This supports the thesis carried out by Garcia et al. where they propose an undertreatment of chronic pain in Latin America [19].

Ruiz-Iban et al. also described insufficient pain treatment for chronic pain in osteoarthritis in Latin America, proposing an underuse of strong opioid analgesics [20]. Thus, the results of this study serve as a baseline regarding the use of opioids in patients with RA.

The relationship found with the use of opioid analgesics for more than 12 months in the patients who were receiving therapy with biological DMARDs may be due to the fact that this group of patients shows a greater activity of the disease and therefore requires analgesic therapy. There are also reports showing that the greater and less-controlled inflammatory activity results in more pain [16, 21]. Therefore, the association with the use of systemic corticosteroids may be related to the increased inflammatory activity in the group of patients with RA who required opioids [10, 22].

Different studies have described the relationship of RA-related pain with depression and anxiety and, therefore, the use of drugs for these clinical conditions [10, 16], which may explain why patients who were receiving antidepressants and benzodiazepines had a higher probability of receiving opioids for more than 12 months. It has been established that depression can increase the sensation and perception of pain, which, when correlated with the activity of RA, can increase the use of opioids [23]. Attending physicians who identify this dual condition should seek adequate, and especially safe, alternatives for effective analgesic management [18, 24–26].

The limitations of this study include those which inherent to an observational study, such as insufficient clinical records on disease activity, the time of RA evolution, or the values of acute phase reactants that could show relationships with the use of opioid analgesics. It was also not possible to determine the particular indication for the use of an opioid (measurements of pain severity are missing, so the opioid indication according to pain intensity could not be assessed) and whether pain originated from a condition other than the joint disease; therefore, it could not be identified if opioids may be used for conditions other than RA in this population nor was it possible to evaluate the potential adverse reactions or problems related to the medications. In addition, it was not possible to confirm opioid, NSAIDs, or acetaminophen purchases outside of the health system or drug dispensing system (NSAIDs and acetaminophen are over-the-counter medications in Colombia). The results of this study can be applied to populations of adult RA patients with similar insurance coverage, and finally, these results may not represent the current prescription patterns of opioids in RA patients. This study does have strengths such as the source of information that has been validated in different studies and the stringency of monitoring of the monthly dispensing of drugs to patients with RA, which ensures that the opioid was delivered directly to the correct person.

## 5. Conclusions

Based on the above findings, it can be concluded that opioid analgesics are frequently used in patients with chronic rheumatic pain, even for periods longer than 12 months, although there is not enough evidence to support the efficacy and safety of opioids. Therefore, prescription of opioids should be well justified and limited to situations that clearly require the drug and, hopefully, for short periods of time. A growing trend in the use of opioids in patients with RA was observed, with a higher proportion of use than in other countries; therefore, it is important to continue studying the reasons for this behavior and apply this knowledge to reducing the risk of undesirable events, especially an overdose, tolerance, and dependence.

## Figures and Tables

**Table 1:** Sociodemographic characteristics and use of opioids by group in a cohort of 1,329 patients with rheumatoid arthritis in Colombia.

Variable	Total patients (*n* = 1329), number (%)	Opioids use patients (*n* = 1129), number (%)	Opioids use < 12 months (*n* = 601), number (%)	Opioids use ≥ 12 months (*n* = 528), number (%)
Age (mean, (SD))	61.8 ± 12.1	61.2 ± 11.8	59.9 ± 12.3	62.6 ± 11.0
Age <45 years	114 (8.6)	107 (9.5)	74 (12.3)	33 (6.3)
Age 45–64.9 years	676 (50.9)	602 (53.3)	322 (53.6)	280 (53.0)
Age ≥ 65 years	539 (40.5)	420 (37.2)	205 (34.1)	215 (40.7)
Female	1103 (83.0)	936 (82.9)	495 (82.3)	441 (83.5)
Weak opioids^*a*^	1102 (82.9)	1102 (97.6)	584 (97.2)	519 (98.3)
Strong opioids^*b*^	167 (12.6)	167 (14.8)	60 (10.0)	107 (20.3)

^*a*^Weak opioids: codeine, dihydrocodeine, hydrocodone, tramadol, and tapentadol. ^*b*^Strong opioids: fentanyl, morphine, oxycodone, hydromorphone, and methadone.

**Table 2:** Patterns of the use of opioids for pain management in a cohort of 1,129 patients with rheumatoid arthritis in Colombia.

Drug	*n* = 1129	(%)	Doses (mean)	Interval (no. of doses/day)	nDDD^*a*^	Therapy duration (months)	Percent female	Mean age (years)
*Weak opioids*								
Codeine	861	76.3	47.7	3	0.47	24.1	0.83	61.5 + 11.6
Tramadol	803	71.1	NA^*∗*^	NA^*∗*^	NA	21.6	0.83	61.5 + 11.6
Hydrocodone	252	22.3	12.9	3	0.86	35.6	0.85	64.9 + 11.2
Tapentadol	9	0.8	109.7	2	0.27	41.3	0.67	64.2 + 11.9

*Strong opioids*								
Oxycodone	76	6.7	32.3	2	0.43	36.0	0.87	64.4 + 12.8
Morphine	74	6.6	NA^*∗*^	NA^*∗*^	NA	35.1	0.81	64.2 + 10.8
Hydromorphone	63	5.6	6.7	1	0.33	36.4	0.86	65.7 + 11.4
Methadone	7	0.6	32.4	3	1.29	53.7	100.00	72.3 + 7.9

^*∗*^Presentation most used in bottle with dropper. ^*a*^Relationship between the mean dose and the defined daily dose. Defined daily dose (DDD): gold standard measuring unit for international drug utilization, monitoring, and research (recommended by the WHO). Each medication has a daily dose defined by the WHO; the calculation presented is the ratio between the average used and the defined daily dose. Being 1, the use of the same dose recommended as DDD.

**Table 3:** Multivariate analysis to identify variables that were associated with a greater probability of receiving opioid analgesics for a period longer than 12 months in a cohort of 1,129 patients with rheumatoid arthritis in Colombia.

Variable	Beta	Sig^*a*^	OR^*b*^	95% CI^*c*^	
				Lower	Upper
Gender: female	−0.018	0.927	0.985	0.71	1.366
Age <45 years	Ref	0.004	Ref	Ref	Ref
Age: 45–64.9 years	0.585	0.014	1.782	1.127	2.819
Age >65 years	0.813	0.001	2.235	1.389	3.598
Treated in Bogotá	−0.249	0.113	0.783	0.579	1.06
Treated in Manizales	0.734	0.01	2.067	1.187	3.596
Acetaminophen	0.189	0.24	1.202	0.884	1.634
Use of antidepressants	0.446	0.001	1.553	1.193	2.021
Use of anticonvulsants	1.192	0.001	3.163	1.606	6.230
Use of benzodiazepines	1.182	0.004	3.203	1.462	7.018
Treated with biological DMARDs	0.422	0.014	1.524	1.09	2.131
Use of systemic corticosteroids	0.474	0.01	1.61	1.121	2.313

^*a*^Significance level. ^*b*^OR: odds ratio. ^*c*^95% confidence interval. DMARDs: disease modifying antirheumatic drugs.

## Data Availability

The data used to support the findings of this study are available from the corresponding author upon request (https://dx.doi.org/10.17504/protocols.io.baneidbe).
